# 2D-cranial T1-black-blood MRI in suspected giant cell arteritis—measurement of vessel wall thickness does not give a diagnostic advantage compared to visual scoring alone

**DOI:** 10.3389/fradi.2025.1597938

**Published:** 2025-07-01

**Authors:** Pascal Seitz, Susana Bucher, Lukas Bütikofer, Britta Maurer, Harald Marcel Bonel, Fabian Lötscher, Luca Seitz

**Affiliations:** ^1^ Department of Rheumatology and Immunology, Inselspital, Bern University Hospital, University of Bern, Bern, Switzerland; ^2^ Department of Clinical Research, CTU Bern, University of Bern, Bern, Switzerland; ^3^ Department of Diagnostic, Interventional and Paediatric Radiology, Inselspital, Bern University Hospital, University of Bern, Bern, Switzerland; ^4^ Campusradiologie, Lindenhofgruppe, Bern, Switzerland

**Keywords:** giant cell arteritis, vasculitis, MRI, vessel wall imaging, T1-black-blood, diagnosis

## Abstract

**Objectives:**

To compare two established scoring schemes for the 2D-T1-weighted “black-blood” MRI sequence (T1-BB) for superficial cranial arteries (SCA) in the diagnosis of giant cell arteritis (GCA).

**Methods:**

Ten arterial segments were evaluated in T1-BB images with two different methods: a visual semiquantitative scheme (T1-BB-VISUAL) and a composite scheme that included both the semiquantitative assessment and a quantitative wall thickness measurement (T1-BB-COMP). The expert clinical diagnosis after ≥6 months of follow-up was the diagnostic reference standard. Diagnostic accuracy and agreement on the segment and patient levels were evaluated for the two different rating schemes.

**Results:**

Retrospectively, 151 consecutive patients with clinically suspected GCA were included. The study cohort consisted of 82 patients with and 69 without GCA. For the T1-BB-COMP and the T1-BB-VISUAL, the sensitivity was 81.7% vs. 87.8% (*p* = 0.025), the specificity was 91.3% vs. 88.4% (*p* = 0.16) and the proportion of correct diagnoses was 86.1% vs. 88.1% (*p* = 0.26), respectively. The overall agreement between the two methods for 1,201 rated arterial segments was very good at 91.6% with a kappa of 0.80. The agreement was higher for segments with a larger calibre than for smaller segments: common superficial temporal arteries 98.0%, occipital arteries 93.2%, frontal branches 89.8% and parietal branches 86.9%. The correlation of wall thickness measurements between readers was strong (Spearman's rho of 0.68). The time needed to apply the T1-BB-VISUAL was about half as long as for the T1-BB-COMP (4.5 vs. 8.95 minutes).

**Conclusion:**

In suspected GCA, the additional measurement of the wall thickness of SCAs in 2D-T1-BB MRI does not lead to a better diagnostic performance compared to visual semiquantitative scoring alone. Visual scoring is preferred due to higher efficiency and reliability.

## Introduction

Giant cell arteritis (GCA) commonly affects the superficial cranial arteries (SCAs) (1). Timely diagnosis and start of treatment are important to prevent complications (1, 2). In all cases of possible GCA, the diagnosis should be confirmed by imaging and/or biopsy (3–6). Current recommendations (at least in a European setting) favour ultrasound of the SCAs as first line modality for the diagnosis of GCA with comparable diagnostic accuracy to magnetic resonance imaging (MRI) but better availability and the possibility to include extracranial vessels such as the axillary arteries. For cranial arteries MRI and FDG-PET-CT can be used as an alternative even though there are more prospective studies with low risk of bias for ultrasound (7). Because imaging needs to be performed as early as possible (especially under glucocorticoids) local availability often defines the diagnostic imaging modality used. Rapid availability of MRI is still restricted to a limited number of centres (7). For MRI of SCAs, a high-resolution, post-contrast, 3-Tesla, fat-suppressed T1-weighted, spin-echo sequence [T1-black-blood (T1-BB)] has proven to be an excellent diagnostic tool (7). The T1-BB has the highest diagnostic accuracy (pooled sensitivity 82%; pooled specificity 92%) for the diagnosis of GCA with the clinical diagnosis as a reference compared to other MR sequences (7–13).

The T1-BB is currently the MRI sequence of choice for suspected GCA of the SCAs and is included in the EULAR recommendations for imaging in large vessel vasculitis (7, 10). However, there are unclear aspects regarding the scoring of this sequence, which may lead to uncertainties in daily clinical practice. Early publications on T1-BB propagated a semiquantitative scoring scheme that involved a combined but purely visual analysis of vessel wall enhancement and thickening with an ordinal scale of 0–3 (≥ 2 considered pathological) (11, 12). In 2014, a prospective landmark study about the T1-BB in suspected GCA combined the prior purely visual assessment scheme with quantitative measurements of vessel wall thickness and included threshold values which had to be met for each score. Again, an ordinal scale of 0–3 was used, with ≥2 considered pathological (13). However, it is not clearly specified how and where to perform these measurements of the vessel wall thickness. Our experience from daily practice shows that the slice and the positioning on the vessel circumference selected for the measurements vary greatly from reader to reader, resulting in large variability of wall thickness measurements. In our experience the purely visual rating scheme is therefore not only much easier to apply, but it also needs considerably less time. In addition, the proposed thresholds for unilateral MRI vessel wall thickness are nearly double the cut-off values used for ultrasound imaging of SCAs in GCA. Also, no distinction is made between the different segments of the temporal artery, which have different diameters on average and different ultrasound cut-off values (14, 15). The two different rating schemes have both been used in parallel in recent years, although it is not known whether they produce comparable results (16, 17). Moreover, in recent meta-analyses, these studies were assessed together (8, 18).

In this retrospective study of 151 patients with clinically suspected GCA, we investigated whether the two different T1-BB assessment schemes yield similar results at the individual patient and arterial segment level. From this we discuss if the additional resources necessary for the combined method are justified.

## Materials and methods

The study was retrospectively conducted in accordance with the Declaration of Helsinki at the University Hospital Bern, Switzerland, a tertiary referral centre for vasculitis. The study was approved by the Ethics Committee Bern, Switzerland (ID: 2021-02169). All patients provided general consent to the further use of their data.

### Study population

The inclusion criteria were as follows: Age ≥50 years; evaluation for suspected GCA; head MRI scan performed between January 1st, 2018, and December 31st, 2021; documented patient's consent. As exclusion criteria were in effect: artifacts precluding image analysis; missing T1-BB; non-GCA vasculitis. The search of hospital records resulted in the identification of 191 consecutive patients; excluded were 40 patients: 35 due to missing T1-BB sequence, 1 due to MR artifacts, 4 due to non-GCA vasculitis. The clinical expert diagnosis ≥6 months after the initial diagnosis was used as the diagnostic reference. Two vasculitis experts (LS, PS, or FL; senior rheumatologists) independently established the reference diagnosis by extensive review of medical records (results from MRI re-reads were not used for this decision). In addition to detailed clinical and laboratory data, the results of two or more diagnostic tests (ultrasound of the TA and axillary arteries; whole body FDG-PET-CT; MRI of the head; biopsy of SCA) were available in 150/151 patients for the determination of the expert diagnosis. The classification into GCA and non-GCA matched for all patients between the two experts.

### Image acquisition

MRI with dedicated head and neck coils with 20 or 64 channels was performed on 3-Tesla scanners (Prisma Fit, Skyra Fit, Verio or Vida; Siemens, Erlangen, Germany); slices were acquired in axial planes and covered the volume from the palate to the vertex (9). The slice thickness of the 3D arterial time-of-flight MR angiography (TOF-MRA) was 0.5 mm (9). The high-resolution, post-contrast, 3-Tesla, fat-suppressed T1-weighted, spin-echo sequence [T1-black-blood (T1-BB)] was acquired after intravenous administration of gadolinium-based contrast agents with the following imaging parameters: acquisition matrix 1,024 × 768; field of view 200 × 200 mm; voxel size 0.260 × 0.195 × 3 mm; TR 500 ms; TE 22 ms; flip angle 70; 30 slices with a slice thickness of 3 mm (12). As contrast agent Gadobutrol (Gadovist, Bayer Healthcare, Berlin, Germany) was used at a dose of 0.1 mmol/kg at the rate of 2 ml/s. The scan time of the T1-BB sequence was 10 minutes and 30 s.

### Image evaluation

Image analysis was performed using a standard reporting workstation with Sectra IDS7 (software version 23.1) by P.S. (10 patients and 20 scans for inter-reader analysis) and L.S. (141 patients), both senior rheumatologists specializing in imaging of vasculitis with 11 and 12 years of experience, respectively. Images were coded before evaluation, i.e., readers were completely blinded to patient data, including the name; only the sex and age of the patients were visible on the images. Ten arterial segments were assessed: posterior auricular artery, occipital artery, common superficial temporal artery (CSTA), frontal and parietal branches of the temporal artery (TA). For each segment, the entire visible length was evaluated. Image brightness could be adjusted to optimize the visualization of the SCA's. The adjustment of the brightness was at the discretion of the rater, no special protocol was used for this. For inter-reader analysis, 20 scans were reread by both readers.

For the evaluation of the time needed to read the images two MRI-scans were randomly chosen per reader, blinded to clinical information. The time for the completion of reading the images and notation of the results for each of up to ten segments was measured in seconds for each MRI-scan. Time measurement was started when TOF and T1-BB where visible together on the screen, ready for reading. Initially the T1-BB-VISUAL scheme was applied. Then, one day later, to limit carry-over effects, the T1-BB-COMP was applied. The average time of the four re-reads per rating scheme was calculated.

### Rating of arteries

**Figure 1:**
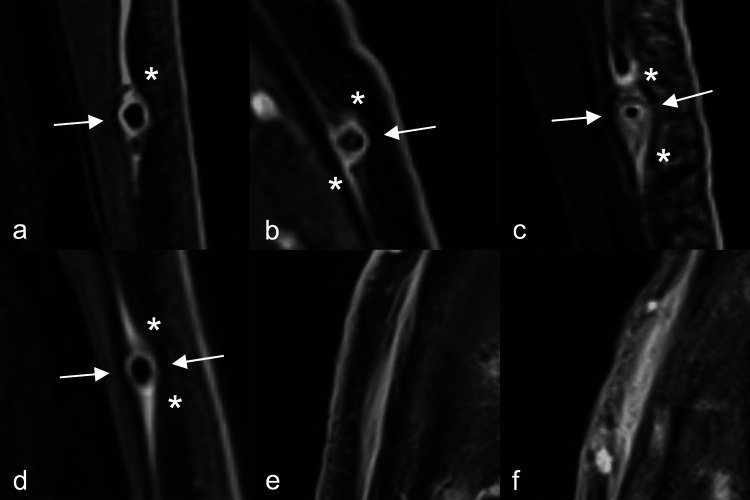
Wall thickness measurements of superficial cranial arteries (all segments pathological). **(a–d)** Arrows indicate where the wall thickness can be measured; asterisks indicate where the wall thickness should not be measured. **(e,f)** if affected frontal branches are running in the axial plane the black blood effect may not work, i.e., the vessel lumen might not be visible (vessel not occluded in corresponding time-of-flight MR angiography); in these cases, measurements of the vessel wall therefore cannot be performed.

The arterial segments were identified with the TOF-MRA on each slice. The T1-BB sequence was rated with two different scoring schemes. For the original scoring scheme (visual semiquantitative, T1-BB-VISUAL), each arterial segment was individually rated with the following scale: 0, no mural thickening and no mural enhancement; 1, no mural thickening with slight mural enhancement; 2, mural thickening with prominent mural enhancement; 3, strong mural thickening with strong mural and perivascular enhancement (11, 12). For examples of this visual scoring scheme, the figure in the original publication can be consulted (11). The absolute value of the vessel wall thickness was not relevant for this scoring scheme, i.e., also segments with a relatively low absolute wall thickness could be assigned a score of 3. T1-BB-VISUAL scores 0 and 1 were considered physiological and scores 2 and 3 to represent vessel wall inflammation (11, 12). For the newer scoring scheme (composite visual and quantitative wall thickness, T1-BB-COMP), the semiquantitative rating was scored first and only then, the wall thickness was measured at the same location, i.e., the knowledge of the measurement value did not influence the semiquantitative rating. T1-BB-VISUAL and T1-BB-COMP were usually assessed at the same location, but the site of the rating did not have to be identical. The latter was typically the case when more distal parts of a segment were also affected. In this case, the thickness criterion was sometimes not fulfilled, despite the presence of pronounced wall inflammation in relation to the vessel calibre, sometimes even with perivascular changes. Each arterial segment was scored according to the following scale for the T1-BB-COMP: 0, no mural thickening (wall thickness <0.6 mm) and no enhancement; 1, no mural thickening with slight mural enhancement and wall thickness <0.6 mm); 2, mural thickening with prominent mural enhancement and wall thickness ≥0.6 mm; 3, strong mural thickening with strong mural and perivascular enhancement and wall thickness ≥0.7 mm (13). The thresholds for wall thickening had to be met in addition for each category (formulated with “and”), e.g., if the wall was visually enhancing and thickened but was <0.6 mm, a score of 1 resulted (13). For examples of this composite scoring scheme, the figure in the original publication can be consulted (13). T1-BB-COMP scores 0 and 1 were considered physiological and scores 2 and 3 to represent vessel wall inflammation. Figures 1a–d shows where the arterial vessel wall thickness was measured. For each measurement, the image was magnified to correctly place the callipers. Measurements down to one hundredth of a millimetre were registered. For T1-BB-COMP and T1-BB-VISUAL, the MRI scan overall was considered pathological if ≥1 arterial segment with a score of ≥2 was present.

**Figure 2:**
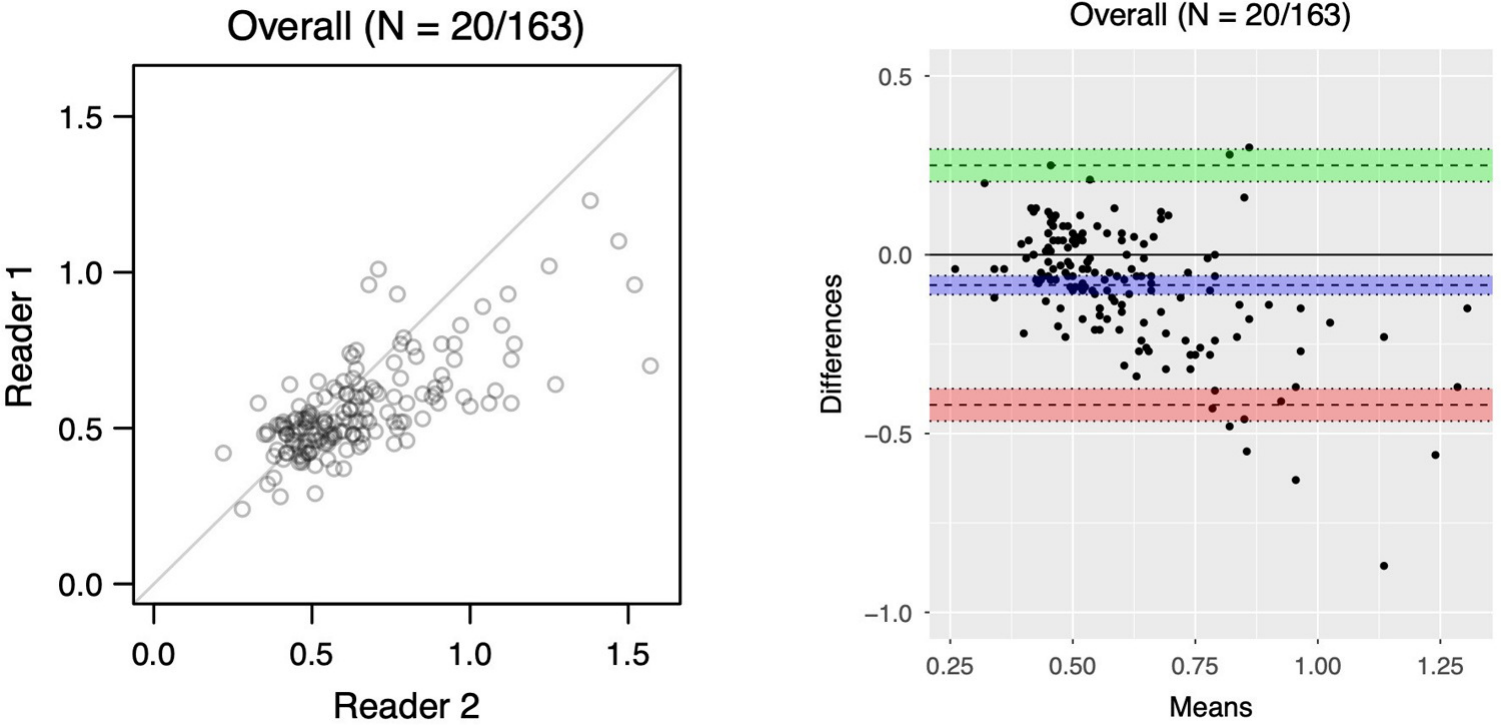
Scatterplot (left) and bland-altman plot (right) of T1-BB wall thickness assessed by two readers for 163 segments from 20 patients. Overall bias of −0.08 mm (95% CI −0.11 to −0.06); lower limit of agreement: −0.42 (95% CI−0.47 to −0.37); upper limit of agreement: 0.25 (95% CI 0.20 to 0.30)).

### Statistics

Statistical analyses were performed with Stata (version 18.0) and the figures were made with R (version 4.3.1) (19, 20). Patient characteristics are reported as absolute and relative frequencies for categorical variables and as median with interquartile range (IQR) for continuous variables. Comparison of baseline characteristics between patient groups was performed using Fisher's exact test and Mann–Whitney-Wilcoxon test for categorical and continuous variables, respectively. The proportion of correct classifications (diagnostic accuracy), sensitivity, specificity and positive and negative predictive values are reported as absolute and relative frequencies with Wilson 95% confidence intervals (CI). Sensitivity, specificity, and the proportion of correct diagnosis were compared between methods using McNemar's test. Positive and negative predictive values were compared using a weighted generalized score statistic, likelihood ratios using a regression model approach, and diagnostic odds ratios using a permutation test (21, 22). Confidence intervals for differences of paired proportions (sensitivity and specificity) were calculated using the Bonett–Price Laplace adjustment. Cohen's kappa with an analytical 95% CI was used to quantify the binary agreement at the patient or segment level. Segment-level correlation of wall thickness measurements was quantified by Spearman's rho with 95% CI (based on Fisher transformation), segment-level agreement was quantified by the intraclass correlation coefficient (ICC).

## Results

Data from 151 patients, 82 (54.3%) with GCA and 69 (45.7%) with different final diagnoses (32 with polymyalgia rheumatica or polyarthritis, 10 with primary headaches, 9 with non-arteritic anterior optic neuropathy, and 18 with other diagnoses), were analysed. Median age was 71 years (IQR 65–77) and 89/151 (58.9%) were female patients. 2022-ACR/EULAR classification criteria for GCA were fulfilled by 79/82 (96.3%) patients with GCA (23). In 123 (81.5%) patients (70 with, 53 without GCA), cranial manifestations were present. Symptoms of polymyalgia rheumatica were present in 84 (55.6%) patients (42 with and 42 without GCA), vision loss in 28 (18.5%; 15 with, 13 without GCA), scalp tenderness in 47 (31.1%; 28 with, 19 without GCA) and jaw claudication in 38 (25.2%; 31 with, 7 without GCA). Median level of C reactive protein was 53 mg/L (IQR 5–111 mg/L) in patients without GCA and 80 mg/L (IQR 46–130 mg/L) in patients with GCA. Glucocorticoids were installed before the MRI in 51/151 (33.8%) patients [27 (32.9%) with GCA; 24 (34.8%) without GCA]; the median duration of glucocorticoid therapy was 0 days (IQR 0–2 days) for all patients. A TA biopsy was performed in 88/151 (58.3%) patients, and it showed vasculitis (detection of inflammatory infiltrate) in 49/88 (59.8%).

### Patient level

Table 1 shows the measures of diagnostic accuracy. We found weak evidence that the sensitivity, negative predictive value and negative likelihood ratio of the T1-BB-COMP could be worse than for T1-BB-VISUAL. No evidence for a difference in specificities, positive predictive values, positive likelihood ratios, the proportion of correct diagnosis (88.1% vs. 86.1%, *P* = 0.26) or the diagnostic odds ratios (54.90 vs. 46.90, *P* = 0.612) was observed. The absolute differences between T1-BB-VISUAL vs. T1-BB-COMP were −2.8% (95% CI −8.2–2.5%) for the specificity, 6.1% (95% CI 0.0–11.9%) for the sensitivity and 2.0% (95% CI −1.8–5.7%) for the proportion of correct diagnosis.

**Table 1:** Diagnostic accuracy compared to the clinical reference diagnosis.

	Abnormal/GCA	Sensitivity (95% CI)	Normal/no GCA	Specificity (95% CI)	PPV (95% CI)	NPV (95% CI)	Positive LR (95% CI)	Negative LR (95% CI)	Correct diagnosis (95% CI)	Diagnostic odds ratio (95% CI)
T1-BB-COMP	67/82	81.7% (72.0–88.6%)	63/69	91.3% (82.3–96.0%)	91.8% (85.7–98.0%)	80.8% (72.0–89.5%)	9.40 (4.34–20.32)	0.20 (0.13–0.32)	130/151 (86.1%, 79.7–90.7%)	46.9 (17.1–128.4)
T1-BB-VISUAL	72/82	87.8% (79.0–93.2%)	61/69	88.4% (78.8–94.0%)	90.0% (83.4–96.6%)	85.9% (77.8–94.0%)	7.57 (3.93–14.60)	0.14 (0.08–0.25)	133/151 (88.1%, 81.9–92.3%)	54.9 (20.4–147.8)
*P*-value^a^		0.025		0.16	0.30	0.041	0.30	0.043	0.26	0.61

^a^ comparison of T1-BB-COMP to T1-BB-VISUAL. PPV, positive predictive value; NPV, negative predictive value; CI, confidence interval; GCA, giant cell arteritis; LR, likelihood ratio; T1-BB: T1-black-blood.

### Segment level

A more nuanced picture emerged by examining the agreement between the two scoring schemes on the segment level. (Table 2) While the overall agreement for 1,201 segments is very good with 91.6% and a kappa of 0.80 (95% CI 0.76–0.84), there was a difference in the total number of segments identified as pathological between the two methods. T1-BB-VISUAL identified 409/1,201 (34.1%) segments as pathological and T1-BB-COMP 308/1,201 (25.6%). Because by definition the visual part of the T1-BB-COMP scoring could not be rated more pathological than the T1-BB-VISUAL, a normal segment in T1-BB-VISUAL could not be pathological in T1-BB-COMP. The binary agreement was better for the segments with larger vessel size (CSTA and occipital arteries) compared to the smaller segments (TA branches and posterior auricular artery).

**Table 2:** Binary agreement between T1-BB-COMP and T1-BB-VISUAL on segment level (*N* = 1,201).

T1-BB-COMP	T1-BB-VISUAL: normal	T1-BB-VISUAL: pathological	Agreement	Cohen's kappa
	*n* (%, 95% CI)	*n* (%, 95% CI)	*n* (%, 95% CI)	(95% CI)
Left CSTA			118 (97.5%, 93.0–99.2%)	0.94 (0.87–1.00)
Normal	85 (100%, 95.7%–100%)	3 (8.3%, 2.9–21.8%)		
Pathological	0 (0.0%, 0.0%–4.3%)	33 (91.7%, 78.2–97.1%)		
Right CSTA			127 (98.4%, 94.5–99.6%)	0.96 (0.91–1.00)
Normal	90 (100%, 95.9%–100%)	2 (5.1%, 1.4–16.9%)		
Pathological	0 (0.0%, 0.0%–4.1%)	37 (94.9%, 83.1–98.6%)		
Left TA frontal branch			132 (90.4%, 84.5–94.2%)	0.79 (0.69–0.89)
Normal	88 (100%, 95.8%–100%)	14 (24.1%, 15.0–36.5%)		
Pathological	0 (0.0%, 0.0%–4.2%)	44 (75.9%, 63.5–85.0%)		
Right TA frontal branch			132 (89.2%, 83.2–93.2%)	0.78 (0.68–0.88)
Normal	81 (100%, 95.5%–100%)	16 (23.9%, 15.3–35.3%)		
Pathological	0 (0.0%, 0.0%–4.5%)	51 (76.1%, 64.7–84.7%)		
Left TA parietal branch			125 (87.4%, 81.0–91.9%)	0.65 (0.51–0.79)
Normal	101 (100%, 96.3%–100%)	18 (42.9%, 29.1–57.8%)		
Pathological	0 (0.0%, 0.0%–3.7%)	24 (57.1%, 42.2–70.9%)		
Right TA parietal branch			126 (86.3%, 79.8–91.0%)	0.66 (0.53–0.79)
Normal	97 (100%, 96.2%–100%)	20 (40.8%, 28.2–54.8%)		
Pathological	0 (0.0%, 0.0%–3.8%)	29 (59.2%, 45.2–71.8%)		
Left post. auricular artery			30 (88.2%, 73.4–95.3%)	0.45 (0.03–0.87)
Normal	28 (100%, 87.9 to 100%)	4 (66.7%, 30.0–90.3%)		
Pathological	0 (0.0%, 0.0–12.1%)	2 (33.3%, 9.7–70.0%)		
Right post. auricular artery			35 (89.7%, 76.4–95.9%)	0.61 (0.28–0.94)
Normal	31 (100%, 89.0%–100%)	4 (50.0%, 21.5–78.5%)		
Pathological	0 (0.0%, 0.0–11.0%)	4 (50.0%, 21.5–78.5%)		
Left occipital artery			139 (94.6%, 89.6–97.2%)	0.87 (0.79–0.96)
Normal	98 (100%, 96.2%–100%)	8 (16.3%, 8.5–29.0%)		
Pathological	0 (0.0%, 0.0%–3.8%)	41 (83.7%, 71.0–91.5%)		
Right occipital artery			136 (91.9%, 86.4–95.3%)	0.82 (0.72–0.92)
Normal	93 (100%, 96.0%–100%)	12 (21.8%, 12.9–34.4%)		
Pathological	0 (0.0%, 0.0–4.0%)	43 (78.2%, 65.6–87.1%)		
Overall			1,100 (91.6%, 89.9–93.0%)	0.80 (0.76–0.84)
Normal	792 (100%, 99.5%–100%)	101 (24.7%, 20.8–29.1%)		
Pathological	0 (0.0%, 0.0%–0.5%)	308 (75.3%, 70.9–79.2%)		

CI, confidence interval; CSTA, common superficial temporal artery; TA, temporal artery.

Table 3 shows the wall thickness for each segment (left and right sides combined). The median and mean of the wall thickness are significantly higher in patients with GCA. While the upper quartiles in No-GCA patients are all below the threshold of 0.6 mm, the value for the CSTA (0.59 mm) is almost identical to it. In No-GCA patients, 15% (84/556) of all segments had a wall thickness of ≥0.6 mm (frontal and parietal branches in 12%–13%; CSTA in 24%). Of these segments, visual analysis of the vessel wall at the same location showed a normal to slightly enhancing wall without thickening in 83% (70/84), i.e., a normal segment according to visual semiquantitative analysis. Accordingly, these segments were classified as normal in T1-BB-COMP, despite a wall thickness above the threshold level. In the GCA subgroup, the wall thickness of the frontal branches is similar to the CSTA and occipital artery, for which the median lies above the threshold of 0.6 mm. The parietal branch and the posterior auricular artery have lower values in comparison.

**Table 3:** Wall thickness on segment level, measured for T1-BB-COMP.

Segment	*N* ^a^	Total	*N* ^a^	No GCA	*N* ^a^	GCA	*P*-value
CSTA	249		117		132		<0.001
Mean (sd)		0.61 (0.16)		0.53 (0.09)		0.67 (0.18)	
Median [lq, uq]		0.57 [0.50, 0.68]		0.54 [0.46, 0.59]		0.63 [0.54, 0.75]	
TA frontal branch	293		133		160		<0.001
Mean (sd)		0.59 (0.18)		0.50 (0.13)		0.66 (0.19)	
Median [lq, uq]		0.54 [0.47, 0.65]		0.49 [0.43, 0.54]		0.63 [0.52, 0.75]	
TA parietal branch	290		133		157		<0.001
Mean (sd)		0.54 (0.16)		0.49 (0.12)		0.58 (0.17)	
Median [lq, uq]		0.51 [0.45, 0.59]		0.48 [0.42, 0.53]		0.54 [0.48, 0.63]	
Post. auricular artery	74		40		34		0.11
Mean (sd)		0.47 (0.12)		0.44 (0.08)		0.50 (0.15)	
Median [lq, uq]		0.44 [0.39, 0.52]		0.44 [0.39, 0.49]		0.47 [0.41, 0.54]	
Occipital artery	293		133		160		<0.001
Mean (sd)		0.59 (0.16)		0.52 (0.09)		0.65 (0.18)	
Median [lq, uq]		0.55 [0.48, 0.67]		0.51 [0.46, 0.56]		0.62 [0.52, 0.73]	

^a^ Number of non-missing observations. CSTA, common superficial temporal artery; GCA, giant cell arteritis; lq, lower quartile; sd, standard deviation; TA, temporal artery; uq, upper quartile.

### Inter-rater analysis

The inter-rater analysis was based on 12 patients with and 8 without GCA and 163 segments (9). For the T1-BB-COMP, the correct diagnosis was given by both readers in 18/20 patients (90%, 95% CI 69.9–97.2%), the binary agreement on the patient level was substantial with 18/20 patients (90.0%, 95% CI 69.9–97.2%), and the binary agreement on the segment level was 80.4% (131/163, 95% CI 73.6–85.7%) with a Cohen's kappa of 0.56 (95% CI 0.43–0.69, moderate agreement) (24). The correlation of vessel wall thickness measurements between readers was strong (Spearman's rho of 0.68, 95% CI 0.58–0.75, for all segments) (25). The inter-reader reliability for wall thickness on the segment level, assessed by intraclass-correlation, showed a moderate reliability of 0.60 (95% CI 0.40–0.73). The Bland-Altmann statistics shows a small bias of −0.08 mm in wall thickness measurements between readers for the 163 segments (Figure 2): the wall thickness measurements of Reader 2, on average, were slightly higher than those from Reader 1. A minority of measurements, mostly of prominently thickened segments, showed a large difference (top right of the scatter plot, bottom right of the Bland-Altmann plot). An inter-rater analysis for T1-BB-VISUAL was previously published by Seitz et al. (see discussion) (9).

### Analysis of time requirements for the reading of MRI scans

Both PS and LS reread 2 MRI scans to evaluate the time needed to apply the two rating schemes. PS needed 264 and 315 s for the T1-BB-VISUAL, 483 and 667 s for the T1-BB-COMP. LS needed 254 and 248 s for the T1-BB-VISUAL, 517 and 481 s for the T1-BB-COMP. On average, for the T1-BB-VISUAL (*n* = 4) 270.3 s (4.5 minutes) and for the T1-BB-COMP (*n* = 4) 537 s (8.95 minutes) were needed. Therefore, it took the readers about twice as long to apply the combined method including vessel wall measurements.

## Discussion

Our study investigated the diagnostic performance of two different SCA scoring schemes for the 2D-T1-BB MRI sequence for suspected GCA. It shows that for experienced readers, the original and easier to apply, semiquantitative and purely visual scoring scheme (T1-BB-VISUAL) is at least as good if not better as the newer method including the quantitative measurement of the arterial vessel wall thickness (T1-BB-COMP). This supports the recently published recommendation by the international Vasculitis Clinical Research Consortium, which was reached through expert consensus, to use the purely visual rating scoring scheme for future research with T1-BB sequences with scientific data (26). Measures of diagnostic accuracy were quite similar between the two methods, which was not unexpected because T1-BB-VISUAL is included in the definition of T1-BB-COMP, for which a single additional criterion, the wall thickness, must be met. With the sequential rating method, the specificity of T1-BB-COMP can only be equal to or higher than T1-BB-VISUAL and the sensitivity of T1-BB-COMP can only be equal to or lower than T1-BB-VISUAL. Given this background, the main question is whether a loss of sensitivity can be compensated by a gain in specificity. We indeed found evidence that the sensitivity of T1-BB-VISUAL (87.8%) may be higher than that of T1-BB-COMP (81.7%, difference: 6.1%, 95% CI 0.0%–11.9%), whereas the specificity of T1-BB-VISUAL (88.4%) may be a little lower than that of T1-BB-COMP (91.3%, difference: −2.8%, 95% CI −8.2%–2.5%). Overall, the difference of the proportion of correct diagnoses between the two methods is 2.0% [88.1% (T1-BB-VISUAL) vs. 86.1% (T1-BB-COMP)] with a 95% CI of −1.8%–5.7% and it is unlikely that the visual method is worse by more than 1.8%, which we do not regard as clinically relevant. Thus, from the standpoint of a clinician performing a diagnostic workup for GCA, the two methods seem to have comparable diagnostic accuracy with slight advantages for the purely visual method in sensitivity. From the perspective of the radiologist reading the MRI scans, the considerable time gain in using the much simpler visual method without compromising on accuracy seems particularly relevant.

It is possible that the observed differences are smaller than they would be in clinical practice because the assessment of the individual segments was strictly sequential for the T1-BB-COMP, i.e., the visual assessment was performed first, followed by the measurement of the wall thickness. Less experienced readers could be misled by a wall thickness above the threshold value to visually judge the corresponding segment as too thick and thus pathological. In such a case, the specificity could be lower than for T1-BB-VISUAL, unlike with our protocol. Since about 15% of all segments from patients without GCA had a wall thickness above the threshold, this could have a relevant impact on specificity, especially for less experienced readers.

Important questions are whether the considerably higher time requirement for wall thickness measurements is justified and how the two scoring schemes perform in detail. While we didn't specifically register the time required for applying the two scoring schemes in all MRI scans, we did analyse a subset where the application of the T1-BB-COMP scheme needed about twice as much time or up to 9 minutes. This large and clinically relevant difference corresponds well to our experience in daily practice. For T1-BB-VISUAL, the slices can simply be scrolled through. With T1-BB-COMP, the image must additionally be magnified for each segment and each measurement, which often needs to be done several times per segment, resulting in up to 8-20 measurements per MRI scan, depending on number of segments available and detail of assessment. The overall agreement between the two methods on a segment level was very good with 91.6% (kappa of 0.80), but there was a 25% difference in the number of segments identified as pathological. Because the agreement for segments with larger diameter was better than for the rest (e.g., 98% for CSTAs), the threshold values may be too high for segments with smaller diameters, such as the parietal branches or posterior auricular arteries. The markedly lower cut-off values for the intima-media thickness in ultrasound, which define different cut-off values for the TA branches than the CSTA, support this assumption (14, 15, 27). In the absence of a diagnostic reference at the segmental level, it is not possible to say how many of the 25% segments were truly segments with vasculitis. Since the overall sensitivity for T1-BB-COMP is a little lower, some segments may have been misclassified due to failure to meet wall thickness thresholds.

Table 3 shows that different arteries and arterial segments do not have the same wall thickness in non-GCA cases. Thus, by using the same threshold values for all SCAs it is more likely that the wall thickness of the CSTA or the occipital artery lies above the threshold than the frontal and parietal branches or the posterior auricular artery. Overall, approximately 15% of all segments in non-GCA cases were above the threshold value of 0.6 mm. The reason why 80% of these segments in non-GCA cases were not classified as pathologic was because visual semiquantitative analysis by the readers classified the segment in the same location as normal. This was probably due to the relative assessment of wall thickness in relation to the local vessel size and the assessment whether the vessel was cut orthogonally at that location.

The inter-rater analysis for the T1-BB-VISUAL was previously reported for the same 20 patients and readers (9). Seitz et al. reported an equal proportion of correct diagnosis and binary agreement on the patient level compared to the T1-BB-COMP in the present study, but the reported level of agreement on the segment level was higher (substantial vs. moderate) (9, 24). This was comparable to recently published data about interrater reliability of the purely visual rating method on the segment level which also holds for non-expert radiologists (28). The difference in wall thickness measurements between readers was very small, especially considering that it was not specified in which of the 30 slices the individual measurements needed to be done. But there was a minority of segments with a surprisingly large difference. The corresponding images were reviewed by both readers together and three main situations associated with larger measurement differences could be identified. For obliquely cut pathological segments with large partial volume effects and for pathological segments with pronounced perivascular enhancement, different measurement locations, i.e., the slice and the position on the wall circumference chosen, lead to differences in the wall thickness between the readers. The third situation involved patients with multiple branches of similar size, i.e., where no clear main bifurcation of the CSTA can be identified but multiple bifurcations are present; in such a situation the two readers sometimes chose different bifurcations resulting in differing nomenclature of the TA segments. Anatomical variations of TAs are a known challenge, especially for follow-up examinations (29). Overall, Reader 1 measured a little more conservatively compared to Reader 2, but the observed difference in measurements between readers did not translate into a difference in proportion of correct diagnoses, which was 90% for both readers for T1-BB-COMP. This may be explained by the assessment of up to ten segments per patient, where small differences in wall thickness measurements would cancel each other out.

Most of the challenges about measuring vessel wall thickness of the SCAs in 2D-T1-BB are related to the fixed axial plane and the slice thickness of 3 mm. The wall thickness is only displayed correctly in segments that run exactly perpendicular to the axial plane. In other situations, partial volume effects often lead to incorrect representations of the true vessel wall thickness with fuzzy borders that can vary depending on brightness and contrast settings. In addition, the proximal and distal tails of the sectioned vessel (Figures 1c,d) often show an apparently increased wall thickness because of the tangentially sliced vessel walls (or perivascular enhancement), whereas the other two walls do not. Sometimes clinicians measure the wall thickness in this area (Figures 1c,d asterisks), which usually results in overestimated wall thickness values and potential misclassifications. Because SCAs, especially CSTAs, often have a very tortuous course and the frontal branches in the temporal region or the proximal occipital arteries typically run almost in the axial plane, several parts of the SCAs are usually not shown in an ideal orthogonal clean cross-section. Measuring vessel wall thickness in a segment that runs in the axial plane is extremely difficult because the segment is often not cut in the centre, and partial volume effects can result in an apparently narrowed or even absent lumen. (Figures 1e,f) Measurement of wall thickness is more easily achieved with ultrasound as longitudinal or transverse images can be obtained by adjusting the orientation of the transducer to the individual course of the arterial segment (15, 30). This would also be possible if a T1-BB sequence was reconstructed three-dimensionally for each SCA individually, with the segment in each slice being cut orthogonally (16). However, while it is technically feasible, this postprocessing is time-consuming and it is yet unclear whether this can be applied in routine clinical practice in every centre with the same image resolution (16). Therefore, 3D-reconstructed MRI sequences might lead to less problems with partial volume but have other drawbacks. At our tertiary center, this technique is not used. It is mentioned as an optional imaging modality in the current EULAR recommendations (7). Especially in the case of obviously pathologic segments, i.e., with perivascular enhancement, measurement of wall thickness seems to be of limited additional benefit. With perivascular enhancement, the resulting apparent wall thickening of the tails can be greatly exaggerated, resulting in “wall thickness” values of sometimes >2 mm if measured incorrectly. Even if the measurement is taken at the correct location on the vessel wall, reliable, accurate and reproducible measurements of the wall thickness remain challenging in such situations.

This study has several limitations. There is a risk of selection bias due to the retrospective design; this was addressed by including every patient with an available MRI and consent in a predefined time span. The proportion of GCA cases is relatively high, most likely due to the study setting at a tertiary university centre. Prospective studies of T1-BB MRI in GCA had similarly high proportions of GCA in their populations (13, 16). The intraindividual comparison between two scoring methods was the focus of this study. Since a selection bias would affect both methods equally, this aspect is less important than if we had examined only one method. It would have been preferable for two readers to re-read all MRI scans; however, due to the significant amount of time required, this was not possible. The individual reasons for each segment with a difference in measurement between readers is difficult to identify. To assess this for every segment was not feasible. There are many possibilities to arrive at different measurements, e.g., choosing different slices for measurements, different placement of the callipers, different interpretation of arterial branching patterns, different brightness settings etc. The pre-specification on which part of the wall to measure (Figures 1a–d) may have influenced the results; this aspect was not specified previously. We decided to do this to obtain at least some standardization of measurements. In our institution, the T1-BB sequence is acquired with 30 slices, and the scanned volume usually includes the vertex, while typically about 20 slices are acquired and the most cranial part of the head with the vessel segments with smaller diameters is not scanned (11–13, 17). That the most distal aspect of an SCA near the vertex is exclusively affected in GCA is extraordinarily rare, and thus we do not believe that this affected the results. Vasculitides other than GCA can rarely affect SCAs, but these were excluded from the study (31, 32). While no formal statement can be made, we would not expect these to behave differently in T1-BB imaging. As there were several different MRI-scanners used, there is a possible influence of scanner type on imaging quality and characteristics. We did not assess for this statistically. Since the two methods were compared on the same images, we do not think that there is an important influence on the reliability of the results.

The uncertainty as to how and where to measure wall thickness, and the problem of partial volume effects with the resulting variability of measurements, make it challenging to apply a scoring method including wall thickness measurements for the 2D T1-BB in clinical practice. In addition, the time required for individual wall thickness measurements of multiple arterial segments is higher than for the visual semiquantitative assessment, an aspect particularly relevant for the busy clinician in daily practice. It is also important to be aware that SCAs, the CSTAs or in particular the occipital arteries, can have a wall thickness of more than 0.6 mm in patients without GCA. In selected cases with borderline findings, an additional, correctly performed measurement of the vessel wall thickness may still be useful for clinical decision making.

In conclusion, purely visual scoring and scoring including the measurement of the vessel wall thickness of SCAs result in comparable diagnostic accuracies for the 2D-T1-BB MRI sequence for suspected GCA for experienced readers. Still, visual scoring alone requires less time and seems more reliable. Because visual semi-quantitative SCA scoring is faster and easier, it may be considered the preferred T1-BB scoring scheme in suspected GCA in daily clinical practice.

## Data Availability

The raw data supporting the conclusions of this article will be made available by the authors, without undue reservation.
